# Efficacy of a Topical Wound Agent Methanesulfonic Acid and Dimethylsulfoxide on In Vitro Biofilms

**DOI:** 10.3390/ijms22179471

**Published:** 2021-08-31

**Authors:** Saskia Schwarzer, Michael Radzieta, Slade O. Jensen, Matthew Malone

**Affiliations:** 1 South West Sydney Limb Preservation and Wound Research, South Western Sydney Local Health District, Sydney 2170, Australia; M.Radzieta@westernsydney.edu.au (M.R.); S.Jensen@westernsydney.edu.au (S.O.J.); matthew.malone@westernsydney.edu.au (M.M.); 2 High Risk Foot Service, Liverpool Hospital, South Western Sydney LHD, Sydney 2170, Australia; 3 Infectious Diseases and Microbiology, School of Medicine, Western Sydney University, Sydney 2170, Australia; 4 Antimicrobial Resistance and Mobile Elements Group, Ingham Institute of Applied Medical Research, Sydney 2170, Australia

**Keywords:** debrichem, biofilm, in vitro, chronic wound, metanesulfonic acid

## Abstract

A topical desiccating wound agent containing methanesulfonic acid, dimethylsulfoxide and amorphous silica was evaluated in three in vitro models for its efficacy against biofilms produced by *Pseudomonas aeruginosa* (ATCC-15442) and *Staphylococcus aureus* (ATCC-6538). The in vitro biofilm models used were; the MBEC Assay^®^, Centre for Disease Control (CDC) Biofilm Reactor^®^ and a Semi-solid biofilm model. A 30-s exposure of a topical wound desiccating agent was used in each model. A complete eradication of viable cells was demonstrated in all models for both strains (*p* < 0.0001). Imaging with scanning electron microscopy (SEM) was performed where possible. All three models demonstrated complete eradication of viable cells with a 30 s application of a topical wound desiccating agent.

## 1. Introduction

Chronic non-healing wounds of the lower extremity represent a global burden to the healthcare system and are a significant contributor to reduced quality of life and morbidity in affected persons. Chronic wounds have typically been classified based on their underlying aetiology with the most commonly reported in the literature being diabetic foot ulcers, venous leg ulcers, Pressure Injuries and non-healing surgical wounds [1]. Despite the underlying pathophysiology differing between these types of chronic wounds they share some common features; most notably prolonged or excessive inflammation [2], persistent infections [3,4], and the inability of dermal and/or epidermal cells to respond to reparative stimuli [5].

Over the last decade there has been significant focus on the role of biofilms as causes of chronic infections and in contributing to delayed wound healing. Biofilms have collectively been described as “aggregates of microorganisms which may be embedded in a protective matrix, may attach to host tissue or in-dwelling medical devices or exist as aggregates in fluids adjacent to those surfaces. In contrast to planktonic microorganisms, biofilm associated microorganisms demonstrate aggregation, reduced growth rates and altered gene expression” [6]. These changes may help to explain behavioural changes and why biofilms can exhibit enhanced tolerance to antimicrobials and the host immune response. In chronic non-healing wounds, evidence suggests that biofilms may be present in up to 80% of cases [7]. Once pathogenic biofilms become established in host tissue, they can drive chronic and persistent infections, which may delay ulcer healing [8].

A significant shift in clinical paradigms has been proposed for the treatment and management of wounds with chronic biofilm infections [9,10]. Physical removal and/or disruption of biofilms by surgical or conservative sharp debridement form the pillar of treatment. However, the spatial distribution of microbial aggregates in tissue [11,12,13] present challenges in ensuring removal, therefore augmenting debridement through the use of topical agents (antimicrobial, non-antimicrobial) has been advocated. A limitation with using topical agents is the limited robust data on their efficacy in vivo. A recent systematic review identified that 90% of all topical wound agents tested for efficacy against biofilm were conducted in vitro, using 16 different models [14]. Although there are acknowledged limitations of in vitro models, such as the absence of a model which truly mimics a human wound, in vitro analysis forms an integral component in screening for potentially beneficial agents.

In this study, we report on a novel topical wound desiccating agent containing metanesulfonic acid (MSA)-dimethylsulfoxide (DMSO) and amorphous silica (SiO_2_) and its efficacy against in vitro biofilms. The use of this gel has been reported in a small case study of lower limb ulcers. The manufacturers of the gel solution report that the use of a desiccating acid as a wound treatment was investigated based on its antibacterial and denaturation effects, and efficacy in other medical areas [15]. DMSO has historically been used in the treatment of ischemic ulcers and scleroderma [16]. We undertook a systematic approach to testing this agent using three separate in vitro biofilm models. The MBEC Assay^®^ is a standardised model for allowing rapid screening of agents for antimicrobial inhibition and efficacy. The CDC Biofilm Reactor^®^ is a standardised in vitro model capable of reproducible biofilms on individual coupons under flow, and the semi-solid model has been designed to simulate a wound environment through the growth of bacteria encapsulated in agar [17].

## 2. Results

### 2.1. Minimum Biofilm Eradication Concentration (MBEC) Assay^®^

**Figure 1:**
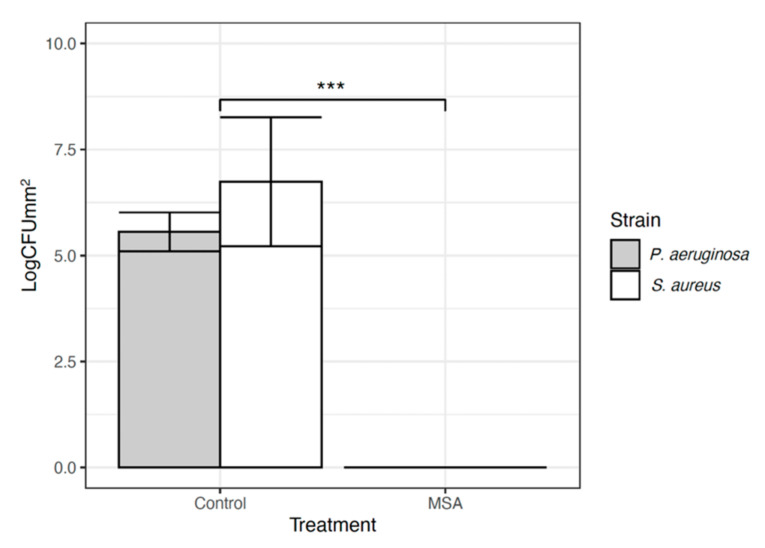
Results for MBEC Assay^®^ for *P. aerurinosa* (grey bar) and *S. aureus* (white bar) reported in CFUmm^2^. The error bars are demonstrating standard deviation. The *** demonstrates statistical significance of *p <* 0.0001.

**Figure 2:**
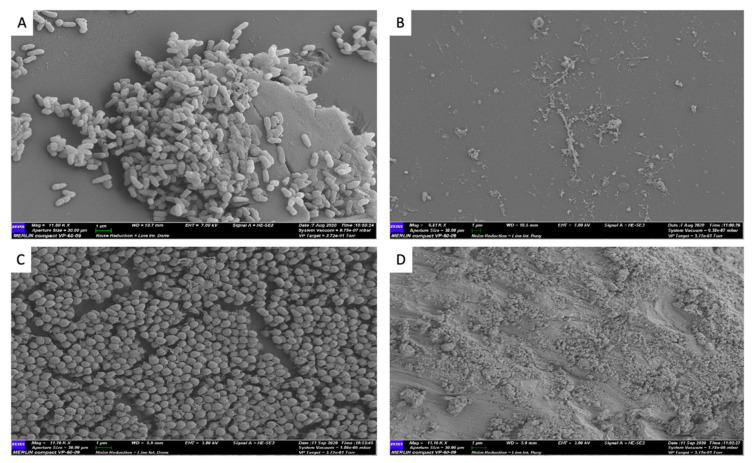
SEM image of *P. aeruginosa* ATCC-15442 biofilm on a MBEC^®^ peg (**A**) control at 11,800 magnification and 1 µm and (**B**) treatment with topical MSA gel solution for 30 s at 6,800 magnification and 1 µm. SEM image of *S. aureus* ATCC-6538 biofilm on a MBEC^®^ peg (**C**) control at 11,700 magnification and 1 µm and (**D**) treatment with topical MSA-DMSO gel solution for 30 s at 11,160 magnification and 1 µm.

A 30-s exposure of topical MSA-DMSO gel solution demonstrated complete eradication of viable cells for American Type Culture Collection (ATCC) *Pseudomonas aeruginosa* (ATCC-15442) and *Staphylococcus aureus* (ATCC-6538) when compared to untreated controls (Figure 1). This equated to a mean log reduction of 5.55 ± 0.4 Log10 CFU/mm^2^ for *P. aeruginosa* (*p* < 0.0001, 95% CI 6.54 to 6.86) and 6.74 ± 1.5 Log10 CFU/mm^2^ for *S. aureus* (*p* < 0.0001, 95% CI 8.53 to 8.83). Scanning Electron Microscopy (SEM) images of untreated control pegs for both *P. aeruginosa and S. aureus* demonstrate the presence of dense microbial aggregates. In contrast, SEM images of pegs treated for 30 s with topical MSA-DMSO gel solution show no microbial aggregates and lysed cells (Figure 2).

### 2.2. CDC Biofilm Reactor^®^

**Figure 3:**
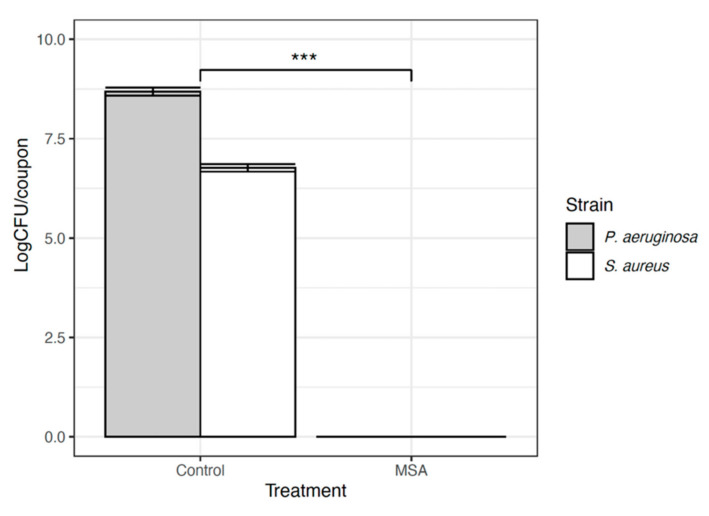
Results from CDC for *P. aeruginosa* and *S. aureus* reported in CFU/coupon. The error bars are demonstrating standard deviation. The *** demonstrates statistical significance of *p <* 0.0001.

**Figure 4:**
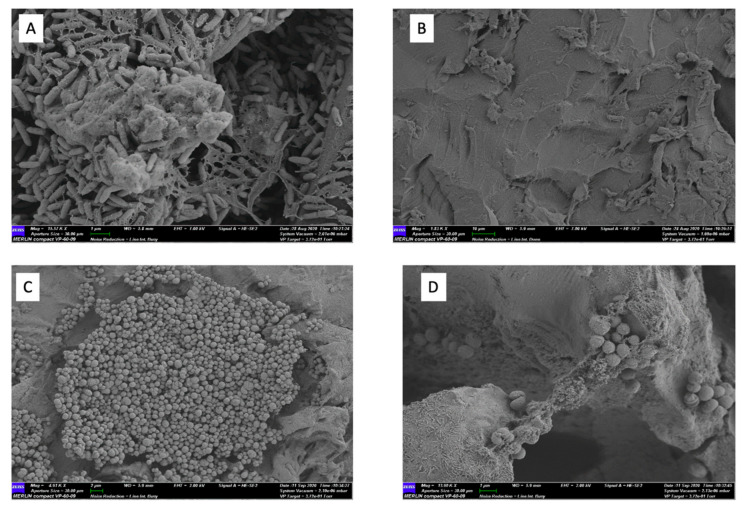
SEM image of *P. aeruginosa* ATCC-15442 biofilm on a polycarbonate coupon (**A**) untreated control at 15,570 magnification and 1 µm and (**B**) treatment with topical MSA-DMSO gel solution for 30 s at 1830 magnification and 1 µm. SEM image of *S. aureus* ATCC-6538 biofilm on a polycarbonate coupon (**C**) Untreated control at 4910 magnification and 2 µm and (**D**) treatment with topical MSA-DMSO gel solution for 30 s at 13,900 magnification and 1 µm.

Polycarbonate discs with *P. aeruginosa* and *S. aureus* biofilms were exposed to a topical MSA-DMSO gel solution for 30 s. When compared against untreated controls, coupons exposed to topical MSA-DMSO gel solution demonstrated complete eradication of viable cells for both *P. aeruginosa* and *S. aureus* (Figure 3). This equated to a reduction of greater than 8.68 ± 0.1 Log10 (*p* < 0.0001, 95% CI 8.5 to 8.3) for *P. aeruginosa* and 6.7 ± 0.1 Log10 (*p* < 0.0001, 95% CI 6.5 to 6.8) for *S. aureus*. SEM images of *P. aeruginosa* and *S. aureus*-untreated coupons show dense aggregates surrounded by extracellular matrix (observed in *P. aeruginosa* only) (Figure 4). No microbial aggregates are seen in treated coupons of *P. aeruginosa* biofilm and small aggregates of <10 bacterial cells are seen in *S. aureus*-treated coupons.

### 2.3. Semi Solid Model

**Figure 5:**
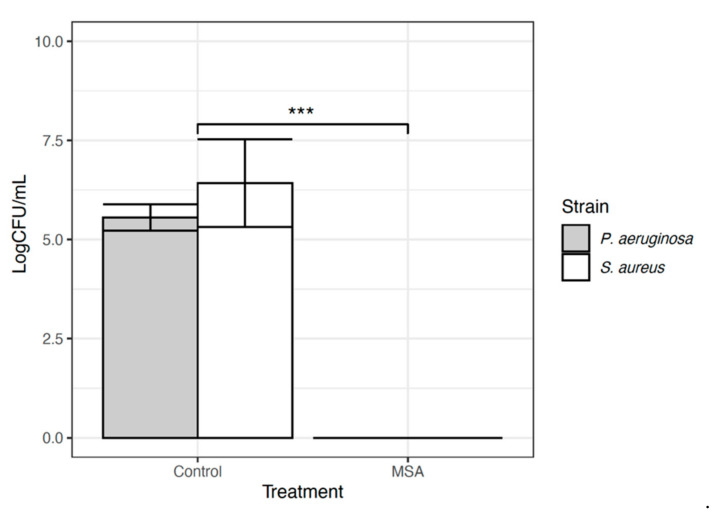
Results from semi solid model for *P. aeuruginosa* and *S. aureus* reported in CFU/mL. The error bars are demonstrating standard deviation. The *** demonstrates statistical significance of *p <* 0.0001.

*P. aeruginosa* and *S. aureus* biofilms encapsulated in a Wound Simulation Medium (WSM) were exposed to a 30 s application to a topical MSA-DMSO gel solution. When compared against untreated control media, treated media demonstrated complete eradication of viable cells for both *P. aeruginosa* (6.07 ± 0.3 Log10, *p* < 0.0001, 95% CI 5.7 to 6.3) and *S. aureus* (6.42 ± 0.3 Log10, *p <* 0.0001, 95% CI 5.4 to 7.4) (Figure 5).

## 3. Discussion

In vitro biofilms are commonly (but not always) embedded in a protective, hydrated, self-produced exopolymeric matrix that comprises lipids, proteins, eDNA and exopolysaccharides [18,19]. Topical agents that induce desiccation through hygroscopy may be beneficial as therapies in managing chronic biofilm infections in humans [9] given their ability to effectively dehydrate components of biofilm architecture and the microbial cells themselves. The agent tested in this series of in vitro models is a novel formulation containing methanesulfonic acid, dimethylsulfoxide and amorphous silica in a gel formulation. The principal action of MSA is its hygroscopicity, a general term used to describe materials that readily take up water from their surroundings. This action, when in proximity to planktonic microbial cells, leads to desiccation of the cell membrane lipid bilayer. This is one of the primary cellular components affected by variations in hydration level, causing changes in lipid packing that may have damaging effects on cell viability [20].

There is some limited preliminary research suggesting that desiccation agents may be an option to consider in managing chronic wounds with biofilm driven infections [21]. Traditional wound care already uses various weak acidic formulations as treatments [22,23] as well as honey which possesses hygroscopic abilities resulting in dehydration. [21,24,25]. These agents exist at low concentrations and are generally well tolerated with low toxicity. There is also evidence to suggest that weak acids could be developed into treatments for biofilm infections, with preliminary studies suggesting that these acids may eradicate biofilm through penetration of the matrix and cell membrane, which may be a similar mechanism to MSA [26].

MSA is classified as a strong acid and through its potent hygroscopic action demonstrated an ability to eradicate all viable cells of both *S. aureus* and *P. aeruginosa* in all three models. Although it has demonstrated effects against bacterial cells, there are likely also collateral affects to host cells. This has not been investigated as part of this work, and safety studies to determine toxicity against host cells are required moving forward. In this study, we have made efforts to ensure that the results are meaningful by testing the product for the duration it should be used for [27], and testing in a range of models from a simple screening tool to a model designed to try and emulate a wound environment.

This study has several limitations to acknowledge. In vitro studies are not reflective of the wound environment [14] and can at best provide indications to progress with further research. The models were performed in triplicate and run independently, but were not performed in multiple laboratories by independent researchers [28]. These models were run as single species, which is not indicative of the polymicrobial nature of chronic wounds [29,30] and clinical isolates were not examined. Safety testing for toxicity to host cells is required.

Although these preliminary investigations have produced promising results, significant further investigation and research is required. The in vitro results indicate that the progression of testing and development may be warranted, however extensive safety testing is required to ensure the response on host cells and surrounding tissue is fully understood and the risks identified, particularly with the known cytotoxic actions of MSA [31,32].

## 4. Materials and Methods

### 4.1. Bacteria

ATCC strains of *P. aeruginosa* (ATCC-15442) (Manassas, VA, USA) and *S. aureus* (ATCC-6538) (Manassas, VA, USA) were used independently in all three models. All broth and agar plates were manufactured in the laboratory from raw materials purchased from Sigma-Aldrich (St. Louis, MI, USA). For the standardised MBEC Assay^®^ and CDC biofilm reactor (BioSurface Technologies Corporation, Bozeman, MT, USA) the strains were streaked on Tryptone Soy Agar (TSA) plates for 24 h at 37 °C and isolated colonies were grown overnight in Tryptone Soy Broth (TSB) at 35 °C and 110 ± 10 rpm. For the semi-solid biofilm model, the strains were streaked on Luria-Bertani (LB) Agar plates was made as described previously and incubated for 24 h at 37 °C. A single colony was then used to inoculate in LB broth which was then incubated overnight at 37 °C and 250 rpm.

### 4.2. Test Agent

Debrichem^®^ (DebX Medical, Amsterdam, The Netherlands) is a topical gel solution with the principal agent being methanesulfonic acid (MSA). MSA acidity is buffered by the use of dimethylsulfoxide (DMSO) and amorphous silica (SiO_2_). The final concentration is 83% MSA and 13.8% DMSO. The gel contains a red dye (1-(p-Nitrophenylazo)-2-naphthol, 1-(4-Nitrophenylazo)-2-naphthol, also known as Para Red) in order to better define the product application on the wound and the surrounding skin. Debrichem is applied topically to the wound bed and the recommended time of application by the manufacturer is 60 s, after which the area is flushed with saline, to cease the action of the acid. For these in vitro experiments, an exposure time of 30 s was selected to 1; ensure application that was less than the maximum application time and 2; to determine if a shorter period of exposure then the currently recommended time provide efficacious. All products used in this study were prepared by DebX Medical, Netherlands.

### 4.3. In Vitro Biofilm Models

All testing was performed in triplicate on two independent occasions.

### 4.4. MBEC Assay^®^

MBEC Assay^®^ testing was completed according to the ASTM International Standard Test Method for Testing Disinfectant Efficacy against *P. aeruginosa* Biofilm using the MBEC Assay^®^ [33]. Briefly, an overnight suspension of each test strain was diluted to 2 × 10^7^ CFU/mL and 1 × 10^7^ CFU/mL for *P. aeruginosa* and *S. aureus*, respectively. A total of 150 µL of each inoculum was added to a 96-well plate (MBEC Assay^®^, Innovotech, Edmonton, AB, Canada), and the 96-peg lid was then inserted onto the plate and incubated for 24 h at 35 ± 2 °C with continuous shaking (110 ± 10 rpm).

Biofilm pegs were washed in sterile saline (NaCl 0.9%) for 10 s, then transferred to a new 96-well plate containing 200 µL of either MSA-DMSO solution or sterile saline (control) for a contact-exposure time of 30 s. Following treatment, the pegs were rinsed twice in saline for 10 s each. At this point, pegs for imaging were removed and fixed in a 4% Glutaraldehyde solution overnight at 4 °C. To facilitate the disaggregation of the biofilm, the recovery plate was sonicated in an ultrasonic water bath (SoniClean™ 160TD, Stepney Australia) on high for 30 min. The recovery solution was then serially diluted, and spot plated in triplicate onto TSA plates and incubated at 35 ± 2 °C for 18–24 h for plate counts and quantitative analysis. In total, nine pegs on each occasion were treated with MSA-DMSO solution for quantification analysis (6 pegs) and SEM imaging (3 pegs), with the same number of control pegs treated with sterile saline (NaCl 0.9%).

### 4.5. CDC Biofilm Reactor^®^ Model

The CDC Biofilm reactor^®^ (BioSurface Technologies Corporation, Bozeman, MT, USA) was prepared using the ASTM Method E3161-18 ‘Standard practice for preparing a *P. aeruginosa* or *S. aureus* Biofilm using the CDC Biofilm Reactor’ [34], and the effect of MSA-DMSO was determined using the ASTM Method E2871-19 ‘Standard Test Method for Determining Disinfectant Efficacy Against Biofilm Grown in the CDC Biofilm Reactor Using the Single Tube Method’20. Briefly, an overnight culture of *P. aeruginosa* was diluted to 2 × 10^7^ CFU/mL, then 1 mL was added to the CDC reactor containing 500 mL sterile 300 mg/L TSB. The reactor was placed on a magnetic stir plate (Talboys™ 4 × 4 Ceramic Stirrer 230 V, USA) at 125 ± 5 r/min at room temperature for a 24-h batch phase growth. A growth medium (100 mg/L TSB) was then added during the continuously stirred tank reactor (CSTR) phase with a residence time of 30 ± 2 min, using a Digital Pump (Masterflex Digital Drive and Pump Head, Vernon Hills, IL, USA) for a further 24 h.

For *S. aureus*, an overnight culture was diluted to 1.2 × 10^7^ CFU/mL before being added to the reactor containing 500 mL of sterile 3 g/L TSB solution. The reactor was placed on the magnetic stir plate at 60 ± 5 r/min and incubated at 36 ± 2 °C for 24 h of batch phase growth. The CSTR phase was then run for an additional 24 h at 36 ± 2 °C using 1 g/L TSB, with a residence time of 30 ± 2 min.

Biofilm coupons of both strains were rinsed in a 50 mL conical centrifuge tube containing 30 mL 0.9% saline. Four coupons were dropped into individual centrifuge tubes containing 4 mL of MSA-DMSO gel solution for 30 s ensuring even coating of the coupons. Control coupons underwent the same treatment process with sterile saline. The coupons stored for imaging were rinsed in 0.9% saline and fixed in 4% Glutaraldehyde solution overnight. Coupons for quantification were then rinsed in a tube containing 10 mL of 0.9% saline and dropped into a new tube containing 36 mL of 0.9% saline. Each tube was then vortexed on the highest setting for 30 ± 5 s at room temperature and then placed in an ultrasonic water bath (SoniClean™, Stepney, South Australia, Australia) at 45 ± 5 kHz for 30 ± 5 s at room temperature. This process was repeated for a total of 3 × rounds of vortexing and 2 × rounds of sonication. For the treated coupons, the entire recovery solution was processed through a Nalgene Reusable Filter Unit with a 0.2 µm filter, with the filter membrane then being placed directly onto a TSA plate. The control coupon tubes were briefly vortexed and diluted from 10^0^ to 10^6^ and plated in duplicate using the spread plate method on TSA. The plates were then incubated at 35 °C for 48 h and 72 h for the control and treated plates, respectively.

### 4.6. Semi Solid Biofilm Model

The semi-solid model has been previously described by Cone et al. [17]. It simulates a wound environment through the growth of bacteria encapsulated in agar.

Briefly, Wound Simulation Medium (WSM) was prepared for a final concentration of; 5 mL Bolton Broth with 1% agar, mixed with 2.5 mL of both bovine fetal serum (Sigma-Aldrich, St Louis, MI, USA) and defibrinated horse blood (Australian Ethical Biologicals, VIC, Australia). Overnight cultures of each strain were grown in LBB and diluted to a final dilution of 10^−4^ in WSM. The semi solid model was assembled by adding WSM to the centre of a gene frame (Thermofisher Scientific, Waltham, MA, USA) adhered to a glass microscope slide (26 mm × 76 mm). The medium was allowed to set before 2 µL of the *P. aeruginosa* and 4 µL of the *S. aureus* inoculum were added to the centre of the medium. Additional WSM was added to encapsulate the inoculum and allowed to set. The slides were then placed in a sterile container with moist tissue paper before being sealed with Parafilm and incubated at 37 °C for 24 h.

Following incubation, 150 µL of MSA-DMSO gel solution was pipetted over the middle of the slide for 30 s, before being rinsed off with approximately 3 mL of sterile saline. Control slides were rinsed with sterile saline. Each gel was then aseptically transferred to a 2 mL Eppendorf tube containing 1.5 mL of sterile saline and a 5 mm stainless steel bead. The tubes then underwent bead beating in a TissueLyser II (Qiagen, Hilden, Germany) at 20 Hz for 15 s to break up the agar. Following bead beating, the contents of each tube were transferred to 45 mL of 0.9% saline and centrifuged at maximum speed (6000× *g*) for 10 min. Following centrifuging, the supernatant was carefully decanted, and the process repeated. All but 2 mL of the supernatant was removed, which was used to resuspend the pellet through vortexing the tube at a low speed. The tubes were then placed in the ultrasonic water bath (SoniClean™, Stepney, South Australia) and degassed for 5 min, followed by sonication on high for 5 min. Each solution was then serially diluted from 10^0^ to 10^6^ in saline, and spot plated in triplicate on LBA plates. The plates were then incubated at 37 ± 2 °C for 18–24 h and colony counted.

### 4.7. Confirmation of Biofilm Growth through Scanning Electron Microscopy (SEM)

SEMwas performed with the LSM 880 confocal microscope (Carl Zeiss, Oberkochen, Germany) to visualise and confirm the growth of bacterial biofilms on the MBEC Assay^®^ pegs and CDC biofilm reactor^®^ polycarbonate discs. Bacterial biofilms were sampled at 5–200 μm with a random field selection. Fixed specimens were dehydrated through serial dilutions of ethanol and hexamethyldisilazane (HDMS) (Polysciences, Inc., Warrington PA, USA). Coupons were rinsed twice in phosphate buffered saline (PBS) then underwent a 50% ethanol and saline wash, 70% ethanol, 95% and two 100% ethanol washes. They were then washed in 2:1 ethanol and HMDS, 1:1, 1:2 ethanol and HMDS and finally two 100% HMDS washes. Coupons were then air dried in the fume hood for at least 48 h. They were then mounted, gold coated for 60 s at a current of 60 mA and examined.

### 4.8. Statistics

Unpaired two-tailed *t* tests were used to compare individual treatments with the control. Data are given as mean, median, and standard deviation (±). Data were analysed through Statistical Package for Social Sciences Version 25 (IBM Corp., Armonk, NY, USA). Data were Log transformed before statistical analyses were performed.

## References

[1] Fife C.E., Eckert K.A., Carter M.J. (2018). Publicly Reported Wound Healing Rates: The Fantasy and the Reality. Adv. Wound Care.

[2] Frykberg R.G., Banks J. (2015). Challenges in the Treatment of Chronic Wounds. Adv. Wound Care.

[3] Jia L., Parker C.N., Parker T.J., Kinnear E.M., Derhy P.H., Alvarado A.M., Huygens F., Lazzarini P.A., Diabetic Foot Working Group, Queensland Statewide Diabetes Clinical Network (2017). Incidence and risk factors for developing infection in patients presenting with uninfected diabetic foot ulcers. PLoS ONE.

[4] McCosker L., Tulleners R., Cheng Q., Rohmer S., Pacella T., Graves N., Pacella R. (2019). Chronic wounds in Australia: A systematic review of key epidemiological and clinical parameters. Int. Wound J..

[5] Demidova-Rice T.N., Hamblin M.R., Herman I.M. (2012). Acute and impaired wound healing: Pathophysiology and current methods for drug delivery, part 1: Normal and chronic wounds: Biology, causes, and approaches to care. Adv. Skin Wound Care.

[6] Cornforth D.M., Dees J.L., Ibberson C.B., Huse H.K., Mathiesen I.H., Kirketerp-Møller K., Wolcott R.D., Rumbaugh K.P., Bjarnsholt T., Whiteley M. (2018). Pseudomonas aeruginosa transcriptome during human infection. Proc. Natl. Acad. Sci. USA.

[7] Malone M., Bjarnsholt T., McBain A.J., James G.A., Stoodley P., Leaper D., Tachi M., Schultz G., Swanson T., Wolcott R.D. (2017). The prevalence of biofilms in chronic wounds: A systematic review and meta-analysis of published data. J. Wound Care.

[8] Metcalf D.G., Bowler P.G. (2013). Biofilm delays wound healing: A review of the evidence. Burn. Trauma.

[9] Schultz G., Bjarnsholt T., James G.A., Leaper D.J., McBain A.J., Malone M., Stoodley P., Swanson T., Tachi M., Wolcott R.D. (2017). Consensus guidelines for the identification and treatment of biofilms in chronic nonhealing wounds. Wound Repair Regen..

[10] Høiby N., Bjarnsholt T., Moser C., Bassi G.L., Coenye T., Donelli G., Hall-Stoodley L., Holá V., Imbert C., Kirketerp-Møller K. (2015). ESCMID guideline for the diagnosis and treatment of biofilm infections 2014. Clin. Microbiol. Infect..

[11] Fazli M., Bjarnsholt T., Kirketerp-Møller K., Jørgensen B., Andersen A.S., Krogfelt K.A., Givskov M., Tolker-Nielsen T. (2009). Nonrandom distribution of Pseudomonas aeruginosa and Staphylococcus aureus in chronic wounds. J. Clin. Microbiol..

[12] Kirketerp-Møller K., Jensen P.Ø., Fazli M., Madsen K.G., Pedersen J., Moser C., Tolker-Nielsen T., Høiby N., Givskov M., Bjarnsholt T. (2008). Distribution, organization, and ecology of bacteria in chronic wounds. J. Clin. Microbiol..

[13] Price L.B., Liu C.M., Frankel Y.M., Melendez J.H., Aziz M., Buchhagen J., Contente-Cuomo T., Engelthaler D.M., Keim P.S., Ravel J. (2011). Macroscale spatial variation in chronic wound microbiota: A cross-sectional study. Wound Repair Regen..

[14] Schwarzer S., James G.A., Goeres D., Bjarnsholt T., Vickery K., Percival S.L., Stoodley P., Schultz G., Jensen S.O., Malone M. (2020). The efficacy of topical agents used in wounds for managing chronic biofilm infections: A systematic review. J. Infect..

[15] Cogo A., Quint B.J., Bignozzi A. (2021). Restarting the Healing Process of Chronic Wounds Using a Novel Dessiccant: A Prospective Case Series. Wounds.

[16] Jacob S.W., Herschler R. (1986). Pharmacology of DMSO. Cryobiology.

[17] Crone S., Garde C., Bjarnsholt T., Alhede M. (2015). A novel in vitro wound biofilm model used to evaluate low-frequency ultrasonic-assisted wound debridement. J. Wound Care.

[18] Whitchurch C.B., Tolker-Nielsen T., Ragas P.C., Mattick J.S. (2002). Extracellular DNA Required for Bacterial Biofilm Formation. Science.

[19] Flemming H.-C., Neu T.R., Wozniak D.J. (2007). The EPS matrix: The “house of biofilm cells”. J. Bacteriol..

[20] Scherber C.M., Schottel J.L., Aksan A. (2009). Membrane phase behavior of Escherichia coli during desiccation, rehydration, and growth recovery. Biochim. Biophys. Acta (BBA) Biomembr..

[21] Park E., Long S., Seth A., Geringer M., Xu W., Chavez-Munoz C., Leung K., Hong S.J., Galiano R.D., Mustoe T.A. (2016). The use of desiccation to treat Staphylococcus aureus biofilm-infected wounds. Wound Repair Regen..

[22] Day A., Alkhalil A., Carney B.C., Hoffman H.N., Moffatt L.T., Shupp J.W. (2017). Disruption of Biofilms and Neutralization of Bacteria Using Hypochlorous Acid Solution: An In Vivo and In Vitro Evaluation. Adv. Skin Wound Care.

[23] Bjarnsholt T., Alhede M., Jensen P.O., Nielsen A.K., Johansen H.K., Homøe P., Høiby N., Givskov M., Kirketerp-Møller K. (2015). Antibiofilm Properties of Acetic Acid. Adv. Wound Care.

[24] Halstead F., Webber M., Rauf M., Burt R., Dryden M., Oppenheim B.A. (2016). In vitro activity of an engineered honey, medical-grade honeys, and antimicrobial wound dressings against biofilm-producing clinical bacterial isolates. J. Wound Care.

[25] Halstead F., Webber M., Oppenheim B. (2017). Use of an engineered honey to eradicate preformed biofilms of important wound pathogens: An in vitro study. J. Wound Care.

[26] Kundukad B., Udayakumar G., Grela E., Kaur D., Rice S.A., Kjelleberg S., Doyle P.S. (2020). Weak acids as an alternative anti-microbial therapy. Biofilm.

[27] Johani K., Malone M., Jensen S.O., Dickson H.G., Gosbell I.B., Hu H., Yang Q., Schultz G., Vickery K. (2018). Evaluation of short exposure times of antimicrobial wound solutions against microbial biofilms: From in vitro to in vivo. J. Antimicrob. Chemother..

[28] Parker A.E., Hamilton M.A., Goeres D.M. (2018). Reproducibility of antimicrobial test methods. Sci. Rep..

[29] Citron D.M., Goldstein E.J., Merriam C.V., Lipsky B.A., Abramson M.A. (2007). Bacteriology of moderate-to-severe diabetic foot infections and in vitro activity of antimicrobial agents. J. Clin. Microbiol..

[30] Johani K., Malone M., Jensen S., Gosbell I., Dickson H., Hu H., Vickery K. (2017). Microscopy visualisation confirms multi-species biofilms are ubiquitous in diabetic foot ulcers. Int. Wound J..

[31] Information NCfB PubChem Compound Summary for CID 6395, Methanesulfonic Acid. https://pubchem.ncbi.nlm.nih.gov/compound/Methanesulfonic-acid.

[32] Bignami M., O’Driscoll M., Aquilina G., Karran P. (2000). Unmasking a killer: DNA O6-methylguanine and the cytotoxicity of methylating agents. Mutat. Res. Rev. Mutat. Res..

[33] ASTM International (2017). E2799-17 Standard Test Method for Testing Disinfectant Efficacy against Pseudomonas Aeruginosa Biofilm Using the MBEC Assay.

[34] ASTM International (2018). E3161-18 Standard Practice for Preparing a Pseudomonas Aeruginosa or Staphylococcus Aureus Biofilm Using the CDC Biofilm Reactor.

